# Publications Are Not the Finish Line: Focusing on Societal Rather Than Publication Impact

**DOI:** 10.3389/fmed.2018.00314

**Published:** 2018-11-12

**Authors:** Farah R. W. Kools, Sara Mirali, Stephanie Holst-Bernal, Sanne L. Nijhof, Giulio Cavalli, Michael A. Grandner

**Affiliations:** ^1^Center of Education and Training, University Medical Center Utrecht, Utrecht University, Utrecht, Netherlands; ^2^Institute of Medical Science, Faculty of Medicine, University of Toronto, Toronto, ON, Canada; ^3^R2C–From Research to Community, Eindhoven, Netherlands; ^4^Department of Pediatrics, Wilhelmina Children's Hospital, University Medical Center Utrecht, Utrecht University, Utrecht, Netherlands; ^5^Unit of Immunology, Rheumatology, Allergy and Rare Diseases, San Raffaele Hospital, Vita-Salute San Raffaele University, Milan, Italy; ^6^Department of Medicine, Radboud University Medical Center, Nijmegen, Netherlands; ^7^Department of Psychiatry, College of Medicine, University of Arizona, Tucson, AZ, United States

**Keywords:** publication, society, impact, accountability, evaluation, translational medicine, interdisciplinary collaboration, scientific waste

## Introduction

Have bibliographical quantification of publications and the subsequent accompanying rewards perverted the incentives of scientists? Are we lost in a publish-or-perish research culture? Alarmingly, ample (bio)medical research findings intended to improve patient outcomes and lead to innovations in patient care never leave the lab (1–3). This widening gap between discovery and implementation undermines the social responsibility of scientists and erodes their public stature. When research findings have the potential to improve the health and well-being of society but are not translated into real-world benefits, it represents a failure of the system and a failure to society.

A re-evaluation of the parameters that define scientific success is imperative. Climbing the academic ladder and securing financial support relies heavily on a scientist's productivity, which is typically defined by the number of publications and their bibliometric scores (4, 5). Several groups are working toward developing novel measures for impact, but so far traditional bibliometric evaluation criteria prevail (6, 7). Whilst understandable that a quantitative system of evaluation might fulfill a desire for objectivity, this creates an intrinsically competitive culture in which regularly publishing ever-novel work is key to individual career success and open collaboration is undermined.

When novel discoveries are incentivized over refinement and implementation, it becomes strategically disadvantageous to do the work needed to translate discoveries into working strategies that benefit patients, the ultimate goal of translational medicine (1–3). Proper recognition and rewards for aiding efforts to achieve this goal must be advocated for, guided by the principles of social accountability and fostered by the support of key stakeholders (8).

## Journals as gatekeepers

One way in which the scientific community is not serving society well is reflected in the current publishing environment. The pressure to publish quantity over quality in order to build a successful scientific career has cultivated a rapidly-expanding ecosystem of thousands of journals publishing millions of papers per year (9). Many of these papers are seldom read or cited, and many contain non-reproducible or even fraudulent data (10, 11). Simultaneously, and partially because of the proliferating abundance of journals, there is increased pressure to publish in so-called “high-impact” journals, which have achieved recognition in the (bio)medical field as being highly desirable to publish in (12–16). Through their selection of what to publish and what not, these “high-impact” journals often become gatekeepers that define what is seen as “good” science by not only the research community, but also the general public. In an effort to impress the editors of these aggrandized journals, scientists increasingly focus on “cutting-edge” questions, rather than validating previous results or pushing them toward further development. Thus, there is a paradoxical problem of too many publications in too many journals, but also too much pressure to publish in too few journals. This creates a conflict where potential scientific advances are lost in the increasingly distracting background noise.

Similar to the role of the free press, scientific journals have a responsibility to the public: to objectively communicate advancements in scientific research and to foster productive exchange of ideas and information. How can journals fulfill this great responsibility? First, by realizing the impact their selection bias has and how strongly it shapes the global scientific research culture. Translational research cannot be accomplished by one individual at a time, it relies heavily on interdisciplinary collaboration and studies at all stages of the research pipeline deserve to be appreciated and rewarded. Second, by helping to shift the focus away from individual achievements and vacuous publication or citation counting, but conversely onto a common goal of achieving real societal impact through collaboration. Encouraging open-access platforms that provide full data sets helps ensure the full use of generated data, reducing scientific waste (17, 18). Web platforms could also implement new evaluation systems, rating scientists on their interdisciplinarity and collaborations. Finally, by revising the peer-review system. Despite holding a very important role in the publishing process, the current system offers little incentive for quality reviewing (19). Unmasking peer-review and rewarding the intellectual contribution and time dedicated by reviewers may promote a more fair process that is in line with the mission of the work. Adding an assessment of the potential for knowledge utilization and societal impact to be published alongside the article would also promote a healthier science culture.

If journals are gatekeepers through which all (bio)medical research must pass, it is time to redefine their role and influence. Translational medicine involves much work beyond initial discovery. The long and tedious but vitally important process of seeing research findings through to clinical practice is one of the field's most overwhelmingly difficult yet largely under-appreciated burdens (20, 21).

## The role of industry, community and other stakeholders

In the case of (bio)medicine, there is a long and risky path from discovery to real-world clinical implementation (22). One research group cannot do all of this alone, especially since the later stages require partnership among many stakeholders (23, 24). If the goal of translational medicine is to implement research that has a meaningful societal impact, academia must collaborate more closely with all stakeholders involved, including industry, patients, and community leaders (6).

A current obstacle to translation is that partnerships among stakeholders are difficult to establish and maintain (25). Specifically, better partnerships between academia and industry would be instrumental to more time- and cost-efficient implementation of research findings (26). Although setting up shared platforms may demand sizeable initial investments, timely and continuing validation of research findings according to companies' pre-approved standards can save time and expenses at later stages of the translation process. More importantly, this facilitates a more efficient pipeline from discovery to societal benefit.

On a more individual scale, Technical Transfer Offices (TTOs), and similar programs housed within academic institutions can also help bridge the gap between academia and industry (27), yet this can be difficult if they are not involved early in the research process and do not remain engaged throughout. Therefore, academic institutions must create awareness amongst scientists and TTOs about their respective value. Specific programs, such as scouting systems to identify potentially impactful research findings, educational initiatives that promote the latest developments, and including TTOs as part of trans-institutional partnerships, might more efficiently establish a pipeline for ideas and networks including international collaborations. Funders could facilitate this by making an assessment of knowledge utilization and societal impact by a third party, e.g., TTO or patient organization, mandatory in annual reports. Sponsored networking events and training programs may also help overcome barriers and facilitate knowledge exchange between these key stakeholders. Developing a more collegial relationship based on shared goals can add momentum to this cooperative process and strengthen the scientific infrastructure as a whole.

Better engagement with other stakeholder groups will facilitate other aspects of the translational enterprise. Patient groups are an increasingly integral part of the scientific process, driving scientific questions (28–30). The voice of the patient in translational research is extremely important and must play a crucial role in the whole process (28). In a similar way, translational medicine has eschewed approaches such as community-based participatory research (CBPR) or community-engaged research (CER) (31, 32). These types of studies, which include community members in the generation of research questions and implementation of research studies, are a valuable approach toward improving the quality and value of the science itself. Involving the community may lead to the identification of underrecognized or underappreciated problems faced by the community, which in turn drives innovation. It may also serve to give a voice to underrepresented and disadvantaged groups that typically fall off the radar. These approaches not only improve scientific validity, innovation, and feasibility, but by including the community as a partner in the work, they kindle a bidirectional dialogue between scientists and society, which is ever more needed.

## Scientific communication

Science in general is facing a growing problem of insufficient resources and eroding public appreciation (33–35). One reason for this is that the public, and funding bodies that often represent the public, are increasingly skeptical about the return on their investment (33, 36). A bench-to-bedside approach to research can help bridge gaps among basic discovery, clinical investigation, implementation, and application in society (37, 38). Effective communication with the public is an important part of this process.

As patients are increasingly confronted by misinformation and charlatanism, the public expresses a desire for clear-cut answers to what they perceive are clear-cut questions. But scientists notoriously provide overly-nuanced and seemingly-obfuscated conclusions. This creates a situation where media reporting of science tends toward overextrapolation and oversimplification which, in turn, leads to scientists being unenthusiastic about engagement with the media or public and the public's distrust of science growing as inaccuracies and exaggerations are borne out, e.g., “miracle cures” that aren't miracles. It is essential that scientists take on their role in guiding the scientific discourse. This is especially true in the field of translational medicine, where discoveries have the potential to directly impact lives.

Communicating science in a way that maintains accuracy, context, and nuance, is accessible to a non-scientific audience, and is as brief as a short news article is difficult, even for seasoned journalists. Additionally, journalists who are expected to cover a wide variety of topics often don't have the expertise or time to assess an individual study's relevance or integrity. It is up to the academics, who have a responsibility to maintain scientific integrity, to accurately interact with the press and advocate for appropriate representation of their work. If academics neglect this role, it will be filled by others who may not hold themselves to the same standards. Yet, scientists are often actively discouraged by peers from collaborating with the media. It is often seen as a distraction or, worse, as unprofessional. Currently though, the ability of scientists to engage the public is greater than it has ever been. More and more news outlets are seeking content, more people than ever are seeking information, and more direct lines of communication are available than there have ever been, e.g., social media.

Issues regarding scientific communication require initiatives at several levels. Academic institutions should better teach scientists how to communicate with the public, ensure that any press releases fairly represent their work, and also powerfully convey relevance to a lay audience. News organizations should collaborate more closely with academia to ensure that reported findings are not overly sensationalized. The public should be encouraged to engage with research with the understanding that while science is rigid in some ways, it reflects a constantly evolving process and an everchanging knowledge base. Improving scientific communication is a critical step in informing everyone, including patients and caregivers, on the relevance and merits of translational medicine. The importance of scientific literacy in communicating the societal impact of research is often and wrongfully neglected.

## Conclusion

Society expects translational scientists to address relevant matters that aim to improve human health and well-being. Indeed, successful translational research has resulted in the clinical application of promising therapies such as CAR-T cell immunotherapy in leukemia and novel HIV antivirals (39, 40). However, the gap between society and academics is widening. Scientists find themselves enthralled in a vicious exercise: publish, secure funding, repeat. The public and other stakeholders are largely absent from this process. Scientists have become so accustomed to this unhealthy system, that they equate “success” with mere survival in the current publish-or-perish culture. Additionally, the perception of science by society and vice versa is dangerously perturbed.

**Figure 1:**
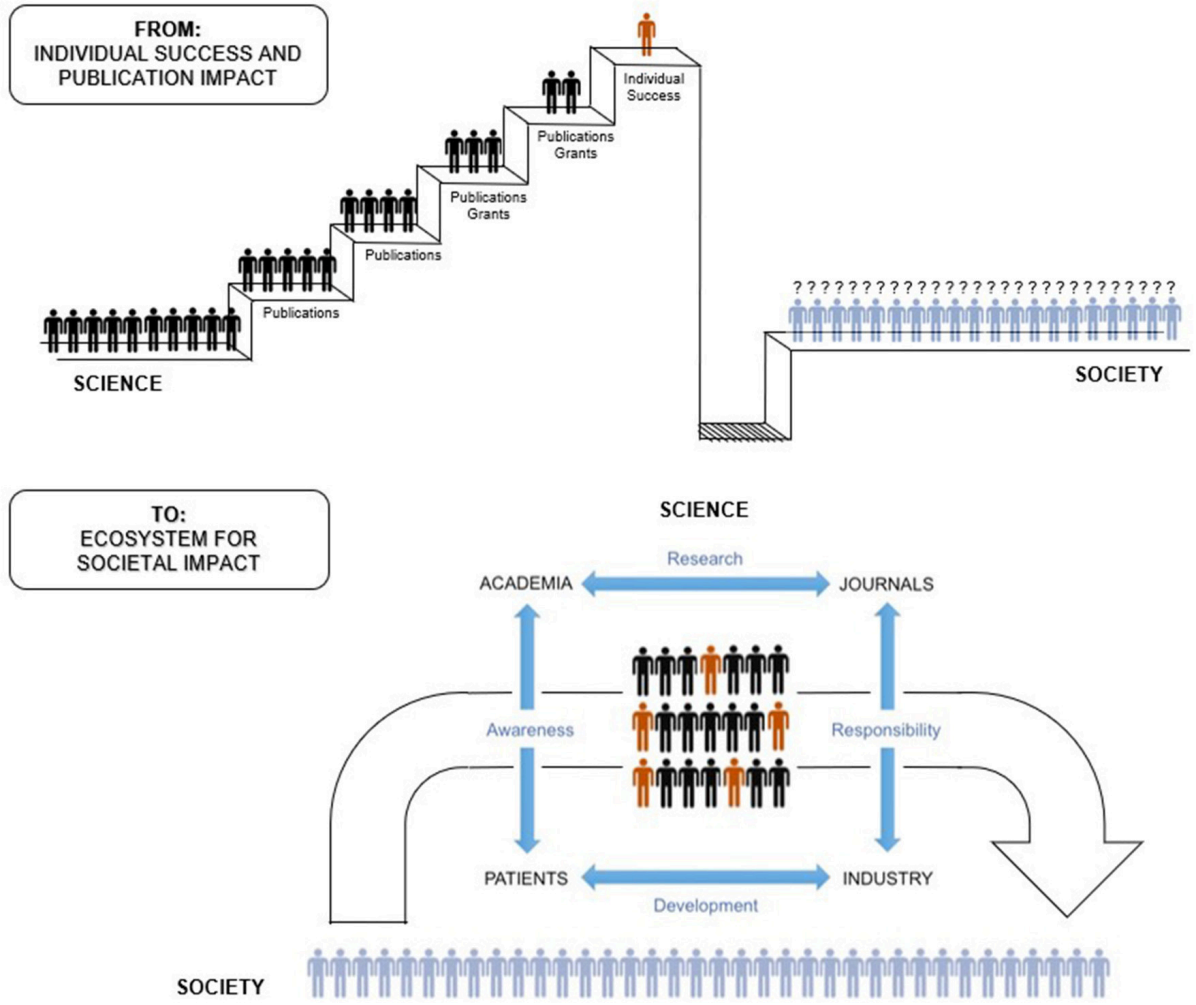
From individual career success and publication impact to a collaborative multidirectional ecosystem for societal impact.

Breaking free from the current failing system will require disrupting this vicious cycle and realigning (bio)medical research with its original mission (Figure 1). This requires reconsideration of the publication system and strategies for including important stakeholders throughout the process. Society must be better informed about the importance of research and play a larger role in its advancement. To accomplish this, scientists and other stakeholders need to take more responsibility in facilitating discussion in a way that effectively communicates and serves the public, while maintaining scientific integrity. Translational scientists should also remember the societal context of their work, recognizing their social accountability and the need for proper two-way dialogue with the public, driving innovation in both directions.

In conclusion, publication should not be the finish line scientists strive to, it should be a stepping stone toward a greater good.

## Author contributions

FK, SM, and SH-B conducted literature research and authored the manuscript. SN and GC advised and guided the writing process. MG revised and edited the final manuscript.

## Conflict of interest statement

The authors declare that the research was conducted in the absence of any commercial or financial relationships that could be construed as a potential conflict of interest. The handling editor declared a shared affiliation, though no other collaboration, with one of the authors FK at time of review.
